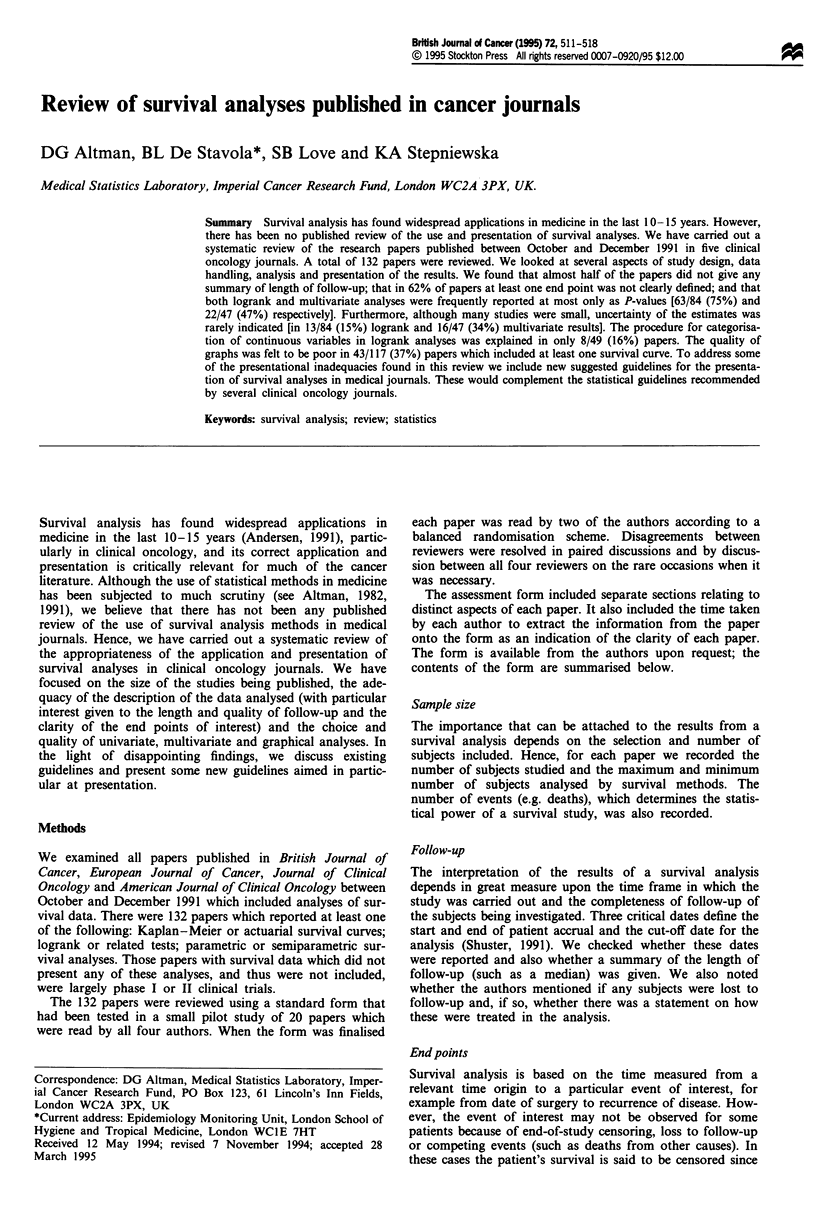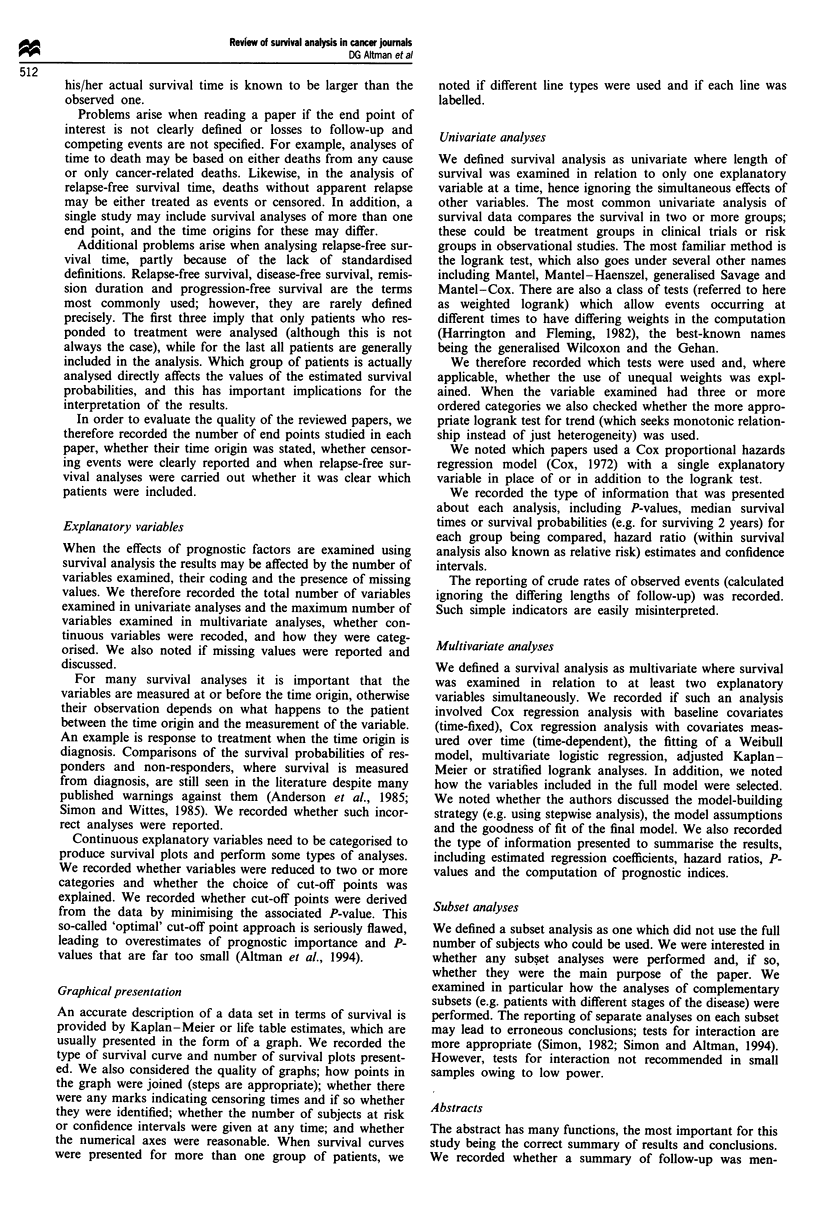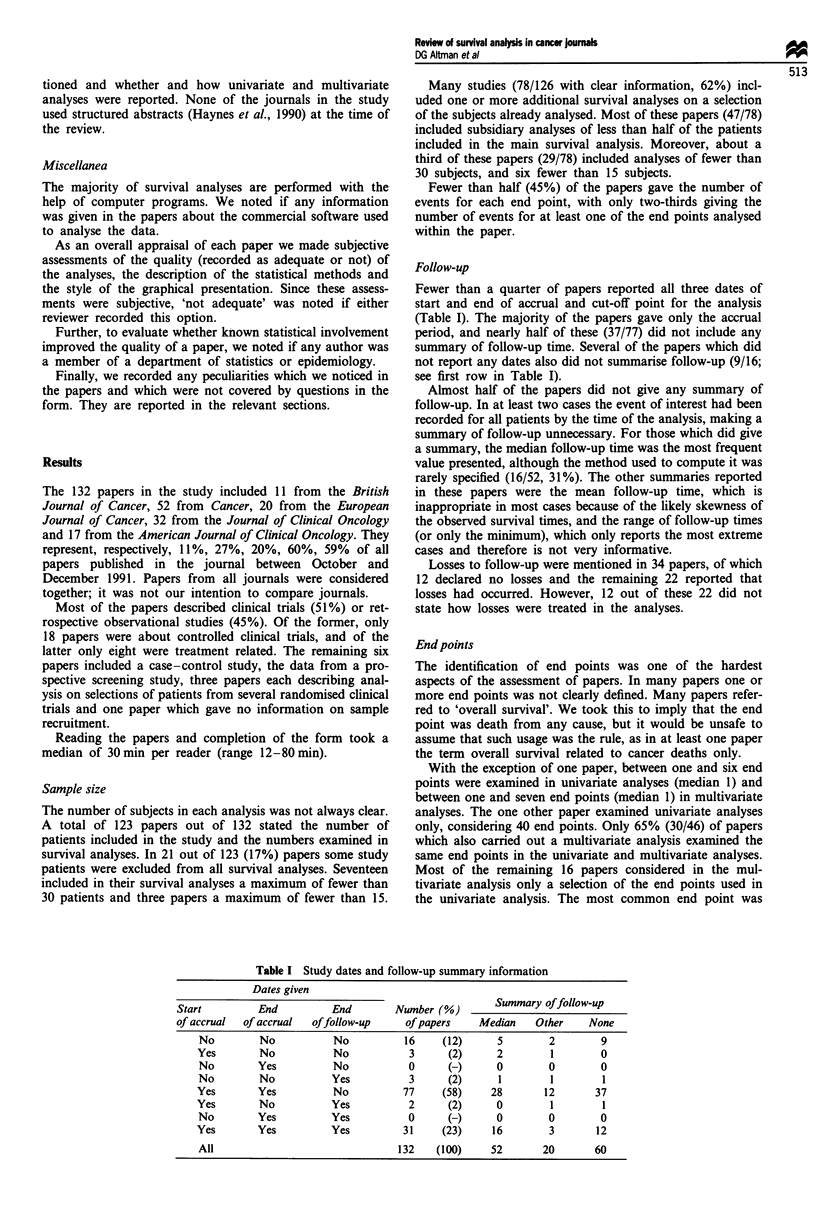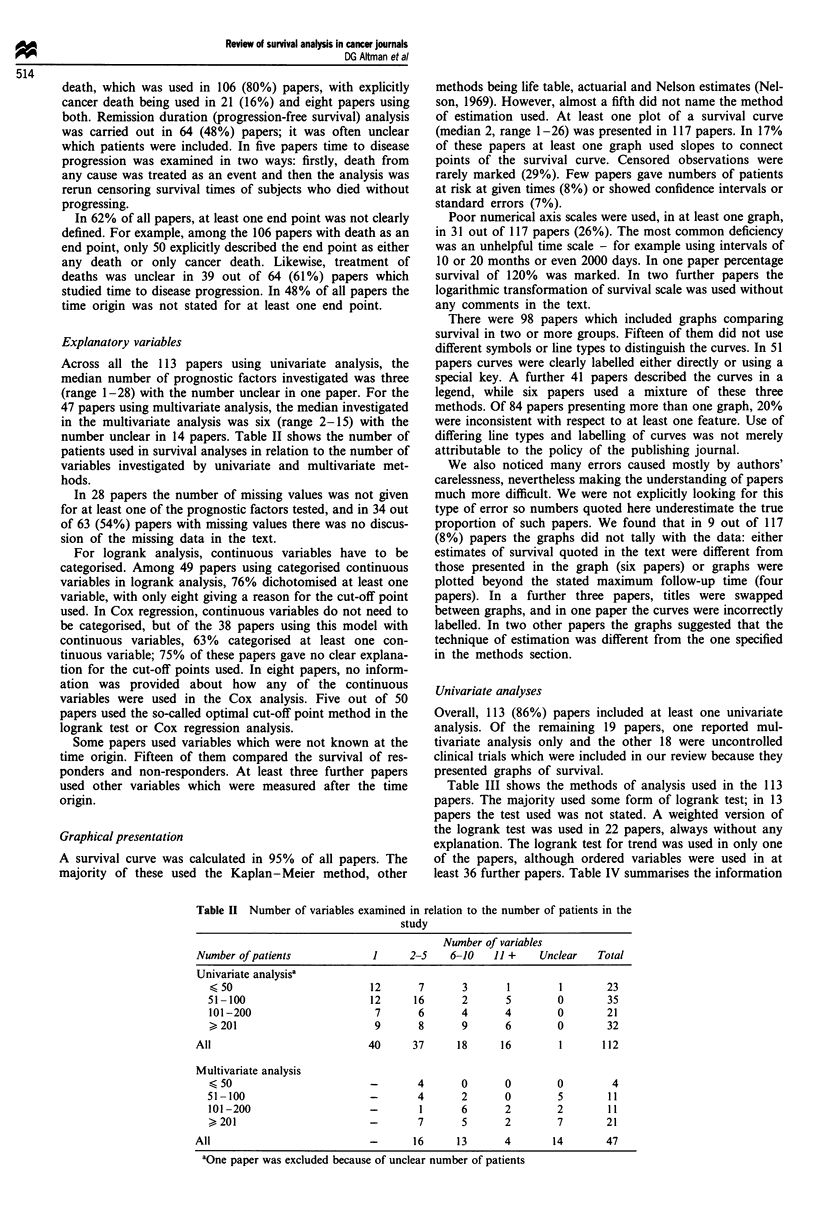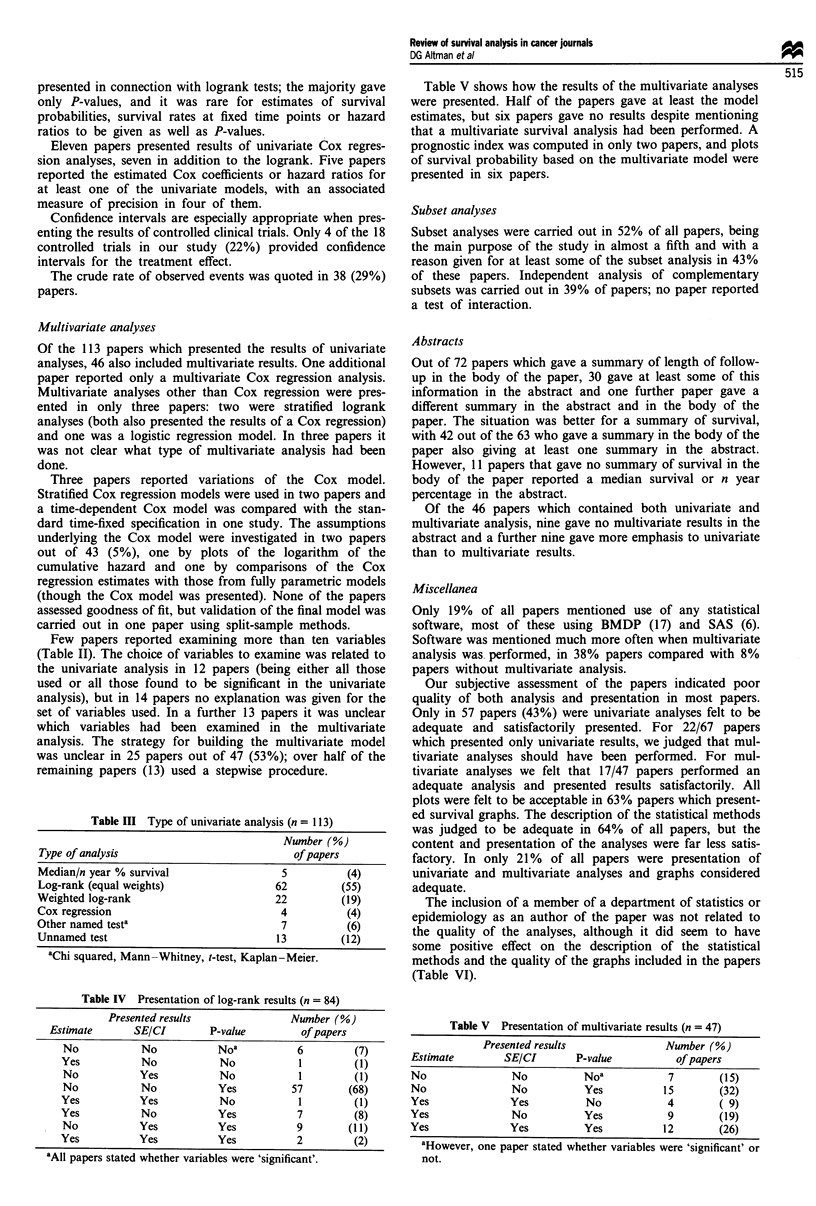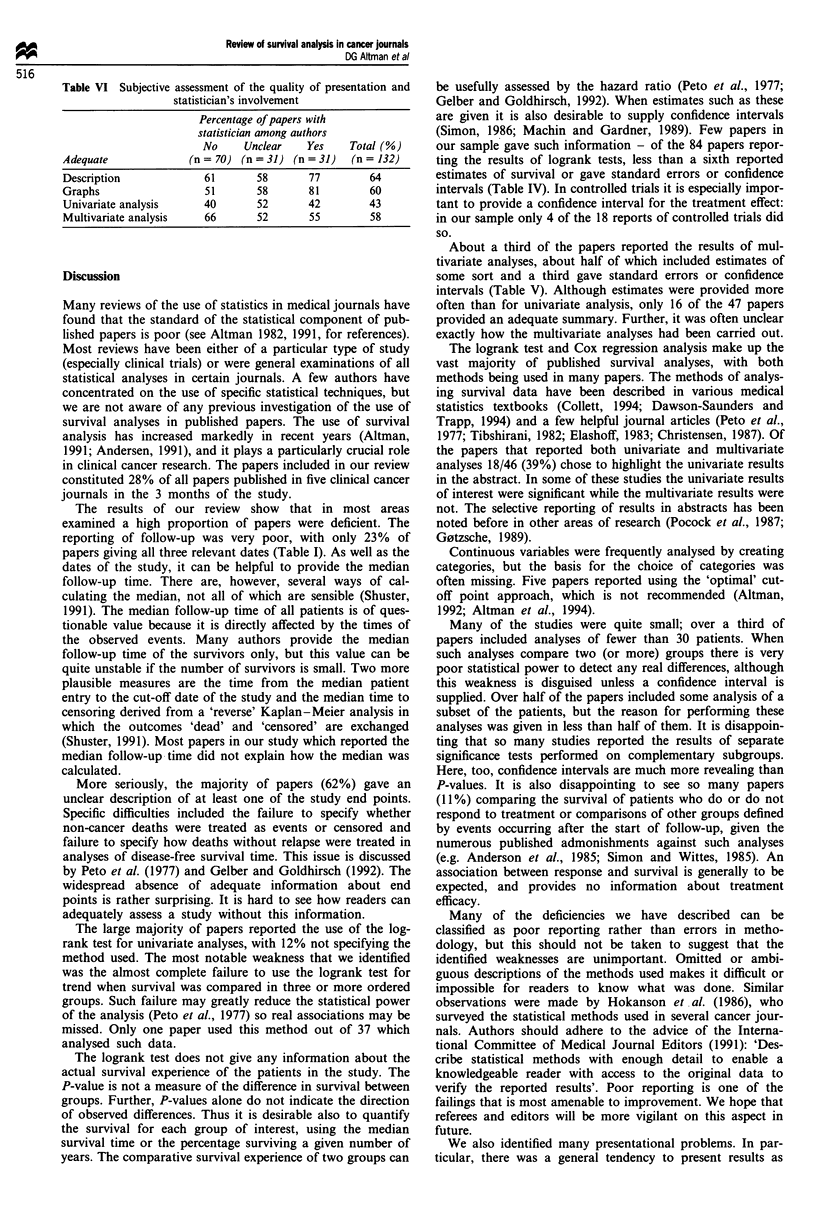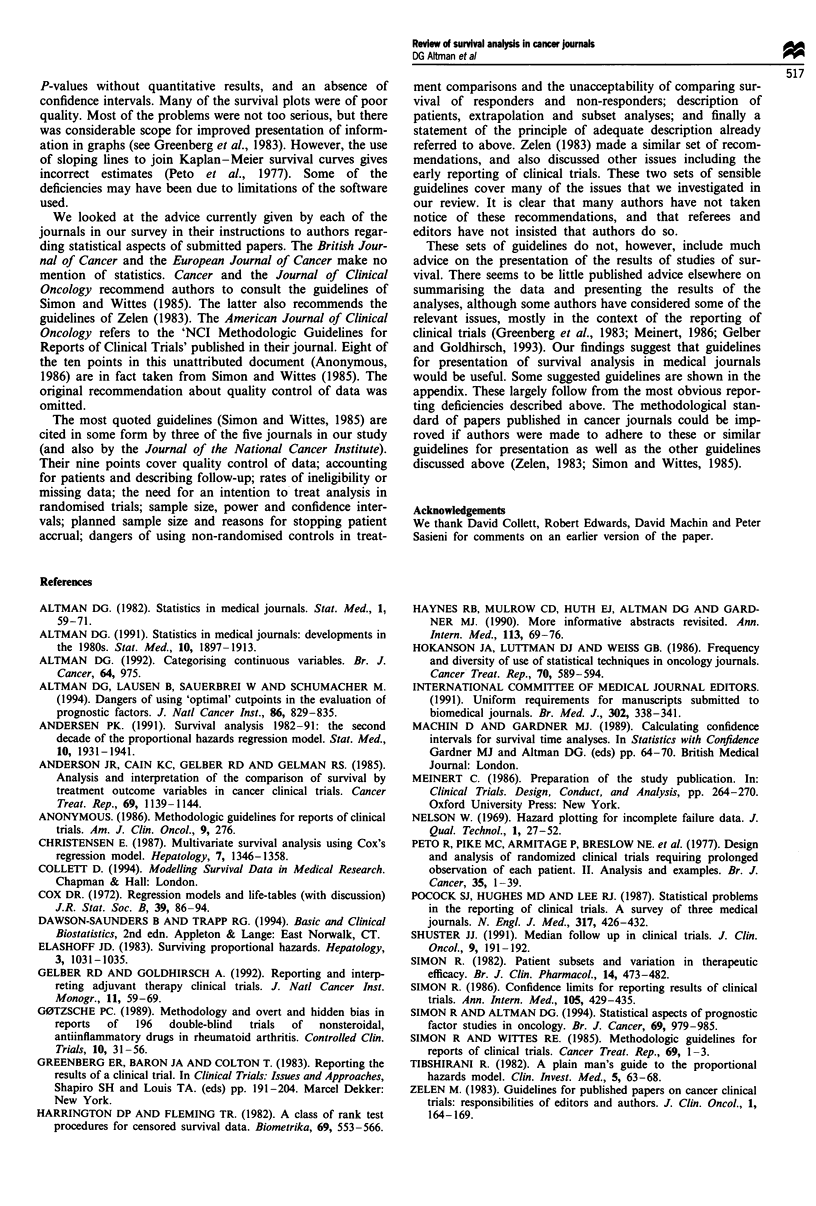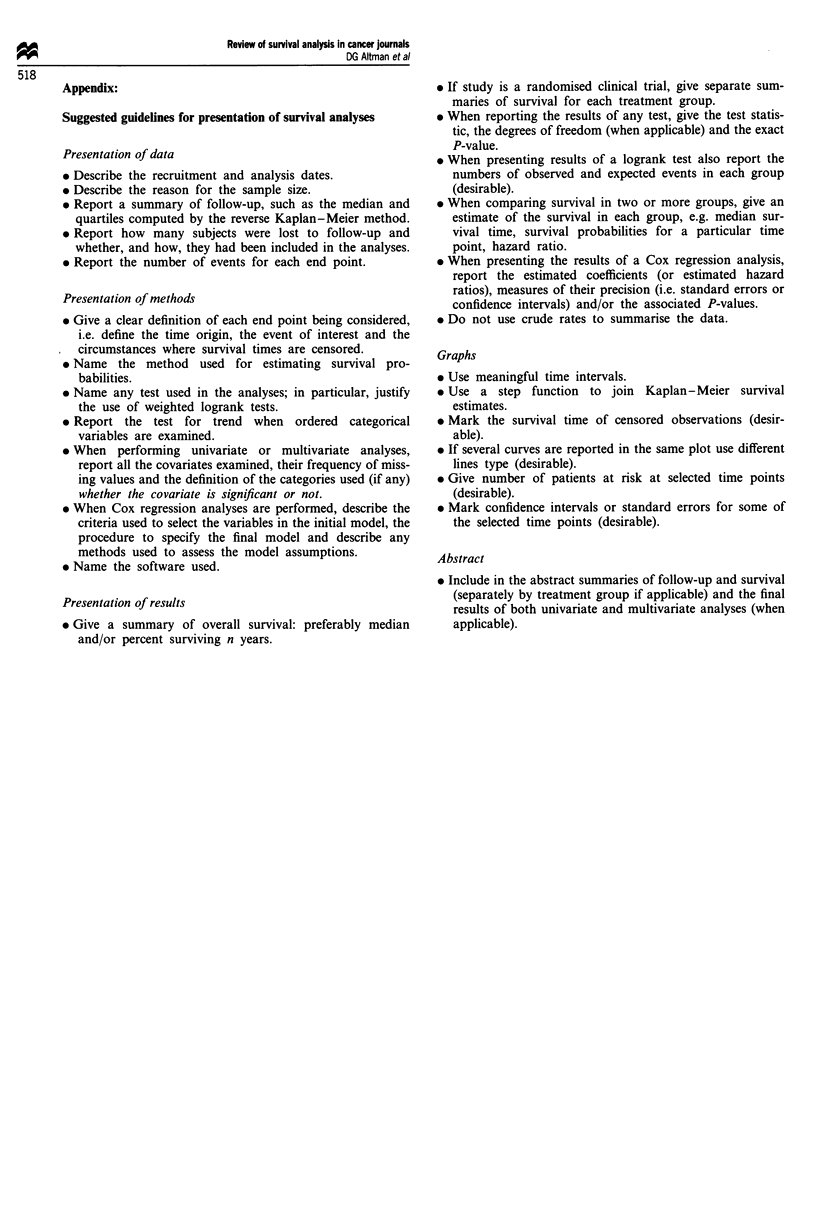# Review of survival analyses published in cancer journals.

**DOI:** 10.1038/bjc.1995.364

**Published:** 1995-08

**Authors:** D. G. Altman, B. L. De Stavola, S. B. Love, K. A. Stepniewska

**Affiliations:** Medical Statistics Laboratory, Imperial Cancer Research Fund, London, UK.

## Abstract

Survival analysis has found widespread applications in medicine in the last 10-15 years. However, there has been no published review of the use and presentation of survival analyses. We have carried out a systematic review of the research papers published between October and December 1991 in five clinical oncology journals. A total of 132 papers were reviewed. We looked at several aspects of study design, data handling, analysis and presentation of the results. We found that almost half of the papers did not give any summary of length of follow-up; that in 62% of papers at least one end point was not clearly defined; and that both logrank and multivariate analyses were frequently reported at most only as P-values [63/84 (75%) and 22/47 (47%) respectively]. Furthermore, although many studies were small, uncertainty of the estimates was rarely indicated [in 13/84 (15%) logrank and 16/47 (34%) multivariate results]. The procedure for categorisation of continuous variables in logrank analyses was explained in only 8/49 (16%) papers. The quality of graphs was felt to be poor in 43/117 (37%) papers which included at least one survival curve. To address some of the presentational inadequacies found in this review we include new suggested guidelines for the presentation of survival analyses in medical journals. These would complement the statistical guidelines recommended by several clinical oncology journals.


British Journal of Cancer (1995) 72, 511–518
© 1995 Stockton Press All rights reserved 0007-0920/95 $12.00

### Review of survival analyses published in cancer journals

DG Altman, BL De Stavola*, SB Love and KA Stepniewska

*Medical Statistics Laboratory, Imperial Cancer Research Fund, London WC2A 3PX, UK.*

**Summary** Survival analysis has found widespread applications in medicine in the last 10–15 years. However, there has been no published review of the use and presentation of survival analyses. We have carried out a systematic review of the research papers published between October and December 1991 in five clinical oncology journals. A total of 132 papers were reviewed. We looked at several aspects of study design, data handling, analysis and presentation of the results. We found that almost half of the papers did not give any summary of length of follow-up; that in 62% of papers at least one end point was not clearly defined; and that both logrank and multivariate analyses were frequently reported at most only as *P*-values [63/84 (75%) and 22/47 (47%) respectively]. Furthermore, although many studies were small, uncertainty of the estimates was rarely indicated [in 13/84 (15%) logrank and 16/47 (34%) multivariate results]. The procedure for categorisation of continuous variables in logrank analyses was explained in only 8/49 (16%) papers. The quality of graphs was felt to be poor in 43/117 (37%) papers which included at least one survival curve. To address some of the presentational inadequacies found in this review we include new suggested guidelines for the presentation of survival analyses in medical journals. These would complement the statistical guidelines recommended by several clinical oncology journals.

**Keywords:** survival analysis; review; statistics

Survival analysis has found widespread applications in medicine in the last 10–15 years (Andersen, 1991), particularly in clinical oncology, and its correct application and presentation is critically relevant for much of the cancer literature. Although the use of statistical methods in medicine has been subjected to much scrutiny (see Altman, 1982, 1991), we believe that there has not been any published review of the use of survival analysis methods in medical journals. Hence, we have carried out a systematic review of the appropriateness of the application and presentation of survival analyses in clinical oncology journals. We have focused on the size of the studies being published, the adequacy of the description of the data analysed (with particular interest given to the length and quality of follow-up and the clarity of the end points of interest) and the choice and quality of univariate, multivariate and graphical analyses. In the light of disappointing findings, we discuss existing guidelines and present some new guidelines aimed in particular at presentation.

### Methods

We examined all papers published in *British Journal of Cancer*, *European Journal of Cancer*, *Journal of Clinical Oncology* and *American Journal of Clinical Oncology* between October and December 1991 which included analyses of survival data. There were 132 papers which reported at least one of the following: Kaplan–Meier or actuarial survival curves; logrank or related tests; parametric or semiparametric survival analyses. Those papers with survival data which did not present any of these analyses, and thus were not included, were largely phase I or II clinical trials.

The 132 papers were reviewed using a standard form that had been tested in a small pilot study of 20 papers which were read by all four authors. When the form was finalised each paper was read by two of the authors according to a balanced randomisation scheme. Disagreements between reviewers were resolved in paired discussions and by discussion between all four reviewers on the rare occasions when it was necessary.

The assessment form included separate sections relating to distinct aspects of each paper. It also included the time taken by each author to extract the information from the paper onto the form as an indication of the clarity of each paper. The form is available from the authors upon request; the contents of the form are summarised below.

### *Sample size*

The importance that can be attached to the results from a survival analysis depends on the selection and number of subjects included. Hence, for each paper we recorded the number of subjects studied and the maximum and minimum number of subjects analysed by survival methods. The number of events (e.g. deaths), which determines the statistical power of a survival study, was also recorded.

### *Follow-up*

The interpretation of the results of a survival analysis depends in great measure upon the time frame in which the study was carried out and the completeness of follow-up of the subjects being investigated. Three critical dates define the start and end of patient accrual and the cut-off date for the analysis (Shuster, 1991). We checked whether these dates were reported and also whether a summary of the length of follow-up (such as a median) was given. We also noted whether the authors mentioned if any subjects were lost to follow-up and, if so, whether there was a statement on how these were treated in the analysis.

### *End points*

Survival analysis is based on the time measured from a relevant time origin to a particular event of interest, for example from date of surgery to recurrence of disease. However, the event of interest may not be observed for some patients because of end-of-study censoring, loss to follow-up or competing events (such as deaths from other causes). In these cases the patient's survival is said to be censored since

Correspondence: DG Altman, Medical Statistics Laboratory, Imperial Cancer Research Fund, PO Box 123, 61 Lincoln's Inn Fields, London WC2A 3PX, UK
*Current address: Epidemiology Monitoring Unit, London School of Hygiene and Tropical Medicine, London WC1E 7HT
Received 12 May 1994; revised 7 November 1994; accepted 28 March 1995

his/her actual survival time is known to be larger than the observed one.

Problems arise when reading a paper if the end point of interest is not clearly defined or losses to follow-up and competing events are not specified. For example, analyses of time to death may be based on either deaths from any cause or only cancer-related deaths. Likewise, in the analysis of relapse-free survival time, deaths without apparent relapse may be either treated as events or censored. In addition, a single study may include survival analyses of more than one end point, and the time origins for these may differ.

Additional problems arise when analysing relapse-free survival time, partly because of the lack of standardised definitions. Relapse-free survival, disease-free survival, remission duration and progression-free survival are the terms most commonly used; however, they are rarely defined precisely. The first three imply that only patients who responded to treatment were analysed (although this is not always the case), while for the last all patients are generally included in the analysis. Which group of patients is actually analysed directly affects the values of the estimated survival probabilities, and this has important implications for the interpretation of the results.

In order to evaluate the quality of the reviewed papers, we therefore recorded the number of end points studied in each paper, whether their time origin was stated, whether censoring events were clearly reported and when relapse-free survival analyses were carried out whether it was clear which patients were included.

*Explanatory variables*

When the effects of prognostic factors are examined using survival analysis the results may be affected by the number of variables examined, their coding and the presence of missing values. We therefore recorded the total number of variables examined in univariate analyses and the maximum number of variables examined in multivariate analyses, whether continuous variables were recoded, and how they were categorised. We also noted if missing values were reported and discussed.

For many survival analyses it is important that the variables are measured at or before the time origin, otherwise their observation depends on what happens to the patient between the time origin and the measurement of the variable. An example is response to treatment when the time origin is diagnosis. Comparisons of the survival probabilities of responders and non-responders, where survival is measured from diagnosis, are still seen in the literature despite many published warnings against them (Anderson *et al.*, 1985; Simon and Wittes, 1985). We recorded whether such incorrect analyses were reported.

Continuous explanatory variables need to be categorised to produce survival plots and perform some types of analyses. We recorded whether variables were reduced to two or more categories and whether the choice of cut-off points was explained. We recorded whether cut-off points were derived from the data by minimising the associated *P*-value. This so-called 'optimal' cut-off point approach is seriously flawed, leading to overestimates of prognostic importance and *P*-values that are far too small (Altman *et al.*, 1994).

*Graphical presentation*

An accurate description of a data set in terms of survival is provided by Kaplan–Meier or life table estimates, which are usually presented in the form of a graph. We recorded the type of survival curve and number of survival plots presented. We also considered the quality of graphs; how points in the graph were joined (steps are appropriate); whether there were any marks indicating censoring times and if so whether they were identified; whether the number of subjects at risk or confidence intervals were given at any time; and whether the numerical axes were reasonable. When survival curves were presented for more than one group of patients, we noted if different line types were used and if each line was labelled.

*Univariate analyses*

We defined survival analysis as univariate where length of survival was examined in relation to only one explanatory variable at a time, hence ignoring the simultaneous effects of other variables. The most common univariate analysis of survival data compares the survival in two or more groups; these could be treatment groups in clinical trials or risk groups in observational studies. The most familiar method is the logrank test, which also goes under several other names including Mantel, Mantel–Haenszel, generalised Savage and Mantel–Cox. There are also a class of tests (referred to here as weighted logrank) which allow events occurring at different times to have differing weights in the computation (Harrington and Fleming, 1982), the best-known names being the generalised Wilcoxon and the Gehan.

We therefore recorded which tests were used and, where applicable, whether the use of unequal weights was explained. When the variable examined had three or more ordered categories we also checked whether the more appropriate logrank test for trend (which seeks monotonic relationship instead of just heterogeneity) was used.

We noted which papers used a Cox proportional hazards regression model (Cox, 1972) with a single explanatory variable in place of or in addition to the logrank test.

We recorded the type of information that was presented about each analysis, including *P*-values, median survival times or survival probabilities (e.g. for surviving 2 years) for each group being compared, hazard ratio (within survival analysis also known as relative risk) estimates and confidence intervals.

The reporting of crude rates of observed events (calculated ignoring the differing lengths of follow-up) was recorded. Such simple indicators are easily misinterpreted.

*Multivariate analyses*

We defined a survival analysis as multivariate where survival was examined in relation to at least two explanatory variables simultaneously. We recorded if such an analysis involved Cox regression analysis with baseline covariates (time-fixed), Cox regression analysis with covariates measured over time (time-dependent), the fitting of a Weibull model, multivariate logistic regression, adjusted Kaplan–Meier or stratified logrank analyses. In addition, we noted how the variables included in the full model were selected. We noted whether the authors discussed the model-building strategy (e.g. using stepwise analysis), the model assumptions and the goodness of fit of the final model. We also recorded the type of information presented to summarise the results, including estimated regression coefficients, hazard ratios, *P*-values and the computation of prognostic indices.

*Subset analyses*

We defined a subset analysis as one which did not use the full number of subjects who could be used. We were interested in whether any subset analyses were performed and, if so, whether they were the main purpose of the paper. We examined in particular how the analyses of complementary subsets (e.g. patients with different stages of the disease) were performed. The reporting of separate analyses on each subset may lead to erroneous conclusions; tests for interaction are more appropriate (Simon, 1982; Simon and Altman, 1994). However, tests for interaction not recommended in small samples owing to low power.

*Abstracts*

The abstract has many functions, the most important for this study being the correct summary of results and conclusions. We recorded whether a summary of follow-up was mentioned and whether and how univariate and multivariate analyses were reported. None of the journals in the study used structured abstracts (Haynes *et al.*, 1990) at the time of the review.

### *Miscellanea*

The majority of survival analyses are performed with the help of computer programs. We noted if any information was given in the papers about the commercial software used to analyse the data.

As an overall appraisal of each paper we made subjective assessments of the quality (recorded as adequate or not) of the analyses, the description of the statistical methods and the style of the graphical presentation. Since these assessments were subjective, 'not adequate' was noted if either reviewer recorded this option.

Further, to evaluate whether known statistical involvement improved the quality of a paper, we noted if any author was a member of a department of statistics or epidemiology.

Finally, we recorded any peculiarities which we noticed in the papers and which were not covered by questions in the form. They are reported in the relevant sections.

### Results

The 132 papers in the study included 11 from the *British Journal of Cancer*, 52 from *Cancer*, 20 from the *European Journal of Cancer*, 32 from the *Journal of Clinical Oncology* and 17 from the *American Journal of Clinical Oncology*. They represent, respectively, 11%, 27%, 20%, 60%, 59% of all papers published in the journal between October and December 1991. Papers from all journals were considered together; it was not our intention to compare journals.

Most of the papers described clinical trials (51%) or retrospective observational studies (45%). Of the former, only 18 papers were about controlled clinical trials, and of the latter only eight were treatment related. The remaining six papers included a case–control study, the data from a prospective screening study, three papers each describing analysis on selections of patients from several randomised clinical trials and one paper which gave no information on sample recruitment.

Reading the papers and completion of the form took a median of 30 min per reader (range 12–80 min).

### *Sample size*

The number of subjects in each analysis was not always clear. A total of 123 papers out of 132 stated the number of patients included in the study and the numbers examined in survival analyses. In 21 out of 123 (17%) papers some study patients were excluded from all survival analyses. Seventeen included in their survival analyses a maximum of fewer than 30 patients and three papers a maximum of fewer than 15.

Many studies (78/126 with clear information, 62%) included one or more additional survival analyses on a selection of the subjects already analysed. Most of these papers (47/78) included subsidiary analyses of less than half of the patients included in the main survival analysis. Moreover, about a third of these papers (29/78) included analyses of fewer than 30 subjects, and six fewer than 15 subjects.

Fewer than half (45%) of the papers gave the number of events for each end point, with only two-thirds giving the number of events for at least one of the end points analysed within the paper.

### *Follow-up*

Fewer than a quarter of papers reported all three dates of start and end of accrual and cut-off point for the analysis (Table I). The majority of the papers gave only the accrual period, and nearly half of these (37/77) did not include any summary of follow-up time. Several of the papers which did not report any dates also did not summarise follow-up (9/16; see first row in Table I).

Almost half of the papers did not give any summary of follow-up. In at least two cases the event of interest had been recorded for all patients by the time of the analysis, making a summary of follow-up unnecessary. For those which did give a summary, the median follow-up time was the most frequent value presented, although the method used to compute it was rarely specified (16/52, 31%). The other summaries reported in these papers were the mean follow-up time, which is inappropriate in most cases because of the likely skewness of the observed survival times, and the range of follow-up times (or only the minimum), which only reports the most extreme cases and therefore is not very informative.

Losses to follow-up were mentioned in 34 papers, of which 12 declared no losses and the remaining 22 reported that losses had occurred. However, 12 out of these 22 did not state how losses were treated in the analyses.

### *End points*

The identification of end points was one of the hardest aspects of the assessment of papers. In many papers one or more end points was not clearly defined. Many papers referred to 'overall survival'. We took this to imply that the end point was death from any cause, but it would be unsafe to assume that such usage was the rule, as in at least one paper the term overall survival related to cancer deaths only.

With the exception of one paper, between one and six end points were examined in univariate analyses (median 1) and between one and seven end points (median 1) in multivariate analyses. The one other paper examined univariate analyses only, considering 40 end points. Only 65% (30/46) of papers which also carried out a multivariate analysis examined the same end points in the univariate and multivariate analyses. Most of the remaining 16 papers considered in the multivariate analysis only a selection of the end points used in the univariate analysis. The most common end point was

**Table I** Study dates and follow-up summary information

| *Dates given* | | | | | *Summary of follow-up* | | |
|---|---|---|---|---|---|---|---|
| *Start of accrual* | *End of accrual* | *End of follow-up* | *Number (%) of papers* | | *Median* | *Other* | *None* |
| No | No | No | 16 | (12) | 5 | 2 | 9 |
| Yes | No | No | 3 | (2) | 2 | 1 | 0 |
| No | Yes | No | 0 | (–) | 0 | 0 | 0 |
| No | No | Yes | 3 | (2) | 1 | 1 | 1 |
| Yes | Yes | No | 77 | (58) | 28 | 12 | 37 |
| Yes | No | Yes | 2 | (2) | 0 | 1 | 1 |
| No | Yes | Yes | 0 | (–) | 0 | 0 | 0 |
| Yes | Yes | Yes | 31 | (23) | 16 | 3 | 12 |
| All | | | 132 | (100) | 52 | 20 | 60 |

death, which was used in 106 (80%) papers, with explicitly cancer death being used in 21 (16%) and eight papers using both. Remission duration (progression-free survival) analysis was carried out in 64 (48%) papers; it was often unclear which patients were included. In five papers time to disease progression was examined in two ways: firstly, death from any cause was treated as an event and then the analysis was rerun censoring survival times of subjects who died without progressing.

In 62% of all papers, at least one end point was not clearly defined. For example, among the 106 papers with death as an end point, only 50 explicitly described the end point as either any death or only cancer death. Likewise, treatment of deaths was unclear in 39 out of 64 (61%) papers which studied time to disease progression. In 48% of all papers the time origin was not stated for at least one end point.

*Explanatory variables*

Across all the 113 papers using univariate analysis, the median number of prognostic factors investigated was three (range 1–28) with the number unclear in one paper. For the 47 papers using multivariate analysis, the median investigated in the multivariate analysis was six (range 2–15) with the number unclear in 14 papers. Table II shows the number of patients used in survival analyses in relation to the number of variables investigated by univariate and multivariate methods.

In 28 papers the number of missing values was not given for at least one of the prognostic factors tested, and in 34 out of 63 (54%) papers with missing values there was no discussion of the missing data in the text.

For logrank analysis, continuous variables have to be categorised. Among 49 papers using categorised continuous variables in logrank analysis, 76% dichotomised at least one variable, with only eight giving a reason for the cut-off point used. In Cox regression, continuous variables do not need to be categorised, but of the 38 papers using this model with continuous variables, 63% categorised at least one continuous variable; 75% of these papers gave no clear explanation for the cut-off points used. In eight papers, no information was provided about how any of the continuous variables were used in the Cox analysis. Five out of 50 papers used the so-called optimal cut-off point method in the logrank test or Cox regression analysis.

Some papers used variables which were not known at the time origin. Fifteen of them compared the survival of responders and non-responders. At least three further papers used other variables which were measured after the time origin.

*Graphical presentation*

A survival curve was calculated in 95% of all papers. The majority of these used the Kaplan–Meier method, other methods being life table, actuarial and Nelson estimates (Nelson, 1969). However, almost a fifth did not name the method of estimation used. At least one plot of a survival curve (median 2, range 1–26) was presented in 117 papers. In 17% of these papers at least one graph used slopes to connect points of the survival curve. Censored observations were rarely marked (29%). Few papers gave numbers of patients at risk at given times (8%) or showed confidence intervals or standard errors (7%).

Poor numerical axis scales were used, in at least one graph, in 31 out of 117 papers (26%). The most common deficiency was an unhelpful time scale – for example using intervals of 10 or 20 months or even 2000 days. In one paper percentage survival of 120% was marked. In two further papers the logarithmic transformation of survival scale was used without any comments in the text.

There were 98 papers which included graphs comparing survival in two or more groups. Fifteen of them did not use different symbols or line types to distinguish the curves. In 51 papers curves were clearly labelled either directly or using a special key. A further 41 papers described the curves in a legend, while six papers used a mixture of these three methods. Of 84 papers presenting more than one graph, 20% were inconsistent with respect to at least one feature. Use of differing line types and labelling of curves was not merely attributable to the policy of the publishing journal.

We also noticed many errors caused mostly by authors' carelessness, nevertheless making the understanding of papers much more difficult. We were not explicitly looking for this type of error so numbers quoted here underestimate the true proportion of such papers. We found that in 9 out of 117 (8%) papers the graphs did not tally with the data: either estimates of survival quoted in the text were different from those presented in the graph (six papers) or graphs were plotted beyond the stated maximum follow-up time (four papers). In a further three papers, titles were swapped between graphs, and in one paper the curves were incorrectly labelled. In two other papers the graphs suggested that the technique of estimation was different from the one specified in the methods section.

*Univariate analyses*

Overall, 113 (86%) papers included at least one univariate analysis. Of the remaining 19 papers, one reported multivariate analysis only and the other 18 were uncontrolled clinical trials which were included in our review because they presented graphs of survival.

Table III shows the methods of analysis used in the 113 papers. The majority used some form of logrank test; in 13 papers the test used was not stated. A weighted version of the logrank test was used in 22 papers, always without any explanation. The logrank test for trend was used in only one of the papers, although ordered variables were used in at least 36 further papers. Table IV summarises the information

**Table II** Number of variables examined in relation to the number of patients in the study

| | *Number of variables* | | | | | |
|---|---|---|---|---|---|---|
| *Number of patients* | *1* | *2–5* | *6–10* | *11 +* | *Unclear* | *Total* |
| Univariate analysis[a] | | | | | | |
| ⩽ 50 | 12 | 7 | 3 | 1 | 1 | 23 |
| 51–100 | 12 | 16 | 2 | 5 | 0 | 35 |
| 101–200 | 7 | 6 | 4 | 4 | 0 | 21 |
| ⩾ 201 | 9 | 8 | 9 | 6 | 0 | 32 |
| All | 40 | 37 | 18 | 16 | 1 | 112 |
| Multivariate analysis | | | | | | |
| ⩽ 50 | – | 4 | 0 | 0 | 0 | 4 |
| 51–100 | – | 4 | 2 | 0 | 5 | 11 |
| 101–200 | – | 1 | 6 | 2 | 2 | 11 |
| ⩾ 201 | – | 7 | 5 | 2 | 7 | 21 |
| All | – | 16 | 13 | 4 | 14 | 47 |

[a]One paper was excluded because of unclear number of patients presented in connection with logrank tests; the majority gave only P-values, and it was rare for estimates of survival probabilities, survival rates at fixed time points or hazard ratios to be given as well as P-values.

Eleven papers presented results of univariate Cox regression analyses, seven in addition to the logrank. Five papers reported the estimated Cox coefficients or hazard ratios for at least one of the univariate models, with an associated measure of precision in four of them.

Confidence intervals are especially appropriate when presenting the results of controlled clinical trials. Only 4 of the 18 controlled trials in our study (22%) provided confidence intervals for the treatment effect.

The crude rate of observed events was quoted in 38 (29%) papers.

*Multivariate analyses*

Of the 113 papers which presented the results of univariate analyses, 46 also included multivariate results. One additional paper reported only a multivariate Cox regression analysis. Multivariate analyses other than Cox regression were presented in only three papers: two were stratified logrank analyses (both also presented the results of a Cox regression) and one was a logistic regression model. In three papers it was not clear what type of multivariate analysis had been done.

Three papers reported variations of the Cox model. Stratified Cox regression models were used in two papers and a time-dependent Cox model was compared with the standard time-fixed specification in one study. The assumptions underlying the Cox model were investigated in two papers out of 43 (5%), one by plots of the logarithm of the cumulative hazard and one by comparisons of the Cox regression estimates with those from fully parametric models (though the Cox model was presented). None of the papers assessed goodness of fit, but validation of the final model was carried out in one paper using split-sample methods.

Few papers reported examining more than ten variables (Table II). The choice of variables to examine was related to the univariate analysis in 12 papers (being either all those used or all those found to be significant in the univariate analysis), but in 14 papers no explanation was given for the set of variables used. In a further 13 papers it was unclear which variables had been examined in the multivariate analysis. The strategy for building the multivariate model was unclear in 25 papers out of 47 (53%); over half of the remaining papers (13) used a stepwise procedure.

**Table III** Type of univariate analysis ($n = 113$)

| *Type of analysis* | *Number (%) of papers* | |
|---|---|---|
| Median/*n* year % survival | 5 | (4) |
| Log-rank (equal weights) | 62 | (55) |
| Weighted log-rank | 22 | (19) |
| Cox regression | 4 | (4) |
| Other named test[a] | 7 | (6) |
| Unnamed test | 13 | (12) |

[a]Chi squared, Mann–Whitney, *t*-test, Kaplan–Meier.

**Table IV** Presentation of log-rank results ($n = 84$)

| *Presented results* | | | *Number (%) of papers* | |
|---|---|---|---|---|
| *Estimate* | *SE/CI* | P-*value* | | |
| No | No | No[a] | 6 | (7) |
| Yes | No | No | 1 | (1) |
| No | Yes | No | 1 | (1) |
| No | No | Yes | 57 | (68) |
| Yes | Yes | No | 1 | (1) |
| Yes | No | Yes | 7 | (8) |
| No | Yes | Yes | 9 | (11) |
| Yes | Yes | Yes | 2 | (2) |

[a]All papers stated whether variables were 'significant'.

Table V shows how the results of the multivariate analyses were presented. Half of the papers gave at least the model estimates, but six papers gave no results despite mentioning that a multivariate survival analysis had been performed. A prognostic index was computed in only two papers, and plots of survival probability based on the multivariate model were presented in six papers.

*Subset analyses*

Subset analyses were carried out in 52% of all papers, being the main purpose of the study in almost a fifth and with a reason given for at least some of the subset analysis in 43% of these papers. Independent analysis of complementary subsets was carried out in 39% of papers; no paper reported a test of interaction.

*Abstracts*

Out of 72 papers which gave a summary of length of follow-up in the body of the paper, 30 gave at least some of this information in the abstract and one further paper gave a different summary in the abstract and in the body of the paper. The situation was better for a summary of survival, with 42 out of the 63 who gave a summary in the body of the paper also giving at least one summary in the abstract. However, 11 papers that gave no summary of survival in the body of the paper reported a median survival or *n* year percentage in the abstract.

Of the 46 papers which contained both univariate and multivariate analysis, nine gave no multivariate results in the abstract and a further nine gave more emphasis to univariate than to multivariate results.

*Miscellanea*

Only 19% of all papers mentioned use of any statistical software, most of these using BMDP (17) and SAS (6). Software was mentioned much more often when multivariate analysis was performed, in 38% papers compared with 8% papers without multivariate analysis.

Our subjective assessment of the papers indicated poor quality of both analysis and presentation in most papers. Only in 57 papers (43%) were univariate analyses felt to be adequate and satisfactorily presented. For 22/67 papers which presented only univariate results, we judged that multivariate analyses should have been performed. For multivariate analyses we felt that 17/47 papers performed an adequate analysis and presented results satisfactorily. All plots were felt to be acceptable in 63% papers which presented survival graphs. The description of the statistical methods was judged to be adequate in 64% of all papers, but the content and presentation of the analyses were far less satisfactory. In only 21% of all papers were presentation of univariate and multivariate analyses and graphs considered adequate.

The inclusion of a member of a department of statistics or epidemiology as an author of the paper was not related to the quality of the analyses, although it did seem to have some positive effect on the description of the statistical methods and the quality of the graphs included in the papers (Table VI).

**Table V** Presentation of multivariate results ($n = 47$)

| *Presented results* | | | *Number (%) of papers* | |
|---|---|---|---|---|
| *Estimate* | *SE/CI* | P-*value* | | |
| No | No | No[a] | 7 | (15) |
| No | No | Yes | 15 | (32) |
| Yes | Yes | No | 4 | ( 9) |
| Yes | No | Yes | 9 | (19) |
| Yes | Yes | Yes | 12 | (26) |

[a]However, one paper stated whether variables were 'significant' or not.

Table VI Subjective assessment of the quality of presentation and statistician's involvement

| | *Percentage of papers with statistician among authors* | | | |
|---|---|---|---|---|
| *Adequate* | *No* (n = *70*) | *Unclear* (n = *31*) | *Yes* (n = *31*) | *Total (%)* (n = *132*) |
| Description | 61 | 58 | 77 | 64 |
| Graphs | 51 | 58 | 81 | 60 |
| Univariate analysis | 40 | 52 | 42 | 43 |
| Multivariate analysis | 66 | 52 | 55 | 58 |

### Discussion

Many reviews of the use of statistics in medical journals have found that the standard of the statistical component of published papers is poor (see Altman 1982, 1991, for references). Most reviews have been either of a particular type of study (especially clinical trials) or were general examinations of all statistical analyses in certain journals. A few authors have concentrated on the use of specific statistical techniques, but we are not aware of any previous investigation of the use of survival analyses in published papers. The use of survival analysis has increased markedly in recent years (Altman, 1991; Andersen, 1991), and it plays a particularly crucial role in clinical cancer research. The papers included in our review constituted 28% of all papers published in five clinical cancer journals in the 3 months of the study.

The results of our review show that in most areas examined a high proportion of papers were deficient. The reporting of follow-up was very poor, with only 23% of papers giving all three relevant dates (Table I). As well as the dates of the study, it can be helpful to provide the median follow-up time. There are, however, several ways of calculating the median, not all of which are sensible (Shuster, 1991). The median follow-up time of all patients is of questionable value because it is directly affected by the times of the observed events. Many authors provide the median follow-up time of the survivors only, but this value can be quite unstable if the number of survivors is small. Two more plausible measures are the time from the median patient entry to the cut-off date of the study and the median time to censoring derived from a 'reverse' Kaplan–Meier analysis in which the outcomes 'dead' and 'censored' are exchanged (Shuster, 1991). Most papers in our study which reported the median follow-up time did not explain how the median was calculated.

More seriously, the majority of papers (62%) gave an unclear description of at least one of the study end points. Specific difficulties included the failure to specify whether non-cancer deaths were treated as events or censored and failure to specify how deaths without relapse were treated in analyses of disease-free survival time. This issue is discussed by Peto *et al.* (1977) and Gelber and Goldhirsch (1992). The widespread absence of adequate information about end points is rather surprising. It is hard to see how readers can adequately assess a study without this information.

The large majority of papers reported the use of the logrank test for univariate analyses, with 12% not specifying the method used. The most notable weakness that we identified was the almost complete failure to use the logrank test for trend when survival was compared in three or more ordered groups. Such failure may greatly reduce the statistical power of the analysis (Peto *et al.*, 1977) so real associations may be missed. Only one paper used this method out of 37 which analysed such data.

The logrank test does not give any information about the actual survival experience of the patients in the study. The *P*-value is not a measure of the difference in survival between groups. Further, *P*-values alone do not indicate the direction of observed differences. Thus it is desirable also to quantify the survival for each group of interest, using the median survival time or the percentage surviving a given number of years. The comparative survival experience of two groups can be usefully assessed by the hazard ratio (Peto *et al.*, 1977; Gelber and Goldhirsch, 1992). When estimates such as these are given it is also desirable to supply confidence intervals (Simon, 1986; Machin and Gardner, 1989). Few papers in our sample gave such information – of the 84 papers reporting the results of logrank tests, less than a sixth reported estimates of survival or gave standard errors or confidence intervals (Table IV). In controlled trials it is especially important to provide a confidence interval for the treatment effect: in our sample only 4 of the 18 reports of controlled trials did so.

About a third of the papers reported the results of multivariate analyses, about half of which included estimates of some sort and a third gave standard errors or confidence intervals (Table V). Although estimates were provided more often than for univariate analysis, only 16 of the 47 papers provided an adequate summary. Further, it was often unclear exactly how the multivariate analyses had been carried out.

The logrank test and Cox regression analysis make up the vast majority of published survival analyses, with both methods being used in many papers. The methods of analysing survival data have been described in various medical statistics textbooks (Collett, 1994; Dawson-Saunders and Trapp, 1994) and a few helpful journal articles (Peto *et al.*, 1977; Tibshirani, 1982; Elashoff, 1983; Christensen, 1987). Of the papers that reported both univariate and multivariate analyses 18/46 (39%) chose to highlight the univariate results in the abstract. In some of these studies the univariate results of interest were significant while the multivariate results were not. The selective reporting of results in abstracts has been noted before in other areas of research (Pocock *et al.*, 1987; Gøtzsche, 1989).

Continuous variables were frequently analysed by creating categories, but the basis for the choice of categories was often missing. Five papers reported using the 'optimal' cut-off point approach, which is not recommended (Altman, 1992; Altman *et al.*, 1994).

Many of the studies were quite small; over a third of papers included analyses of fewer than 30 patients. When such analyses compare two (or more) groups there is very poor statistical power to detect any real differences, although this weakness is disguised unless a confidence interval is supplied. Over half of the papers included some analysis of a subset of the patients, but the reason for performing these analyses was given in less than half of them. It is disappointing that so many studies reported the results of separate significance tests performed on complementary subgroups. Here, too, confidence intervals are much more revealing than *P*-values. It is also disappointing to see so many papers (11%) comparing the survival of patients who do or do not respond to treatment or comparisons of other groups defined by events occurring after the start of follow-up, given the numerous published admonishments against such analyses (e.g. Anderson *et al.*, 1985; Simon and Wittes, 1985). An association between response and survival is generally to be expected, and provides no information about treatment efficacy.

Many of the deficiencies we have described can be classified as poor reporting rather than errors in methodology, but this should not be taken to suggest that the identified weaknesses are unimportant. Omitted or ambiguous descriptions of the methods used makes it difficult or impossible for readers to know what was done. Similar observations were made by Hokanson *et al.* (1986), who surveyed the statistical methods used in several cancer journals. Authors should adhere to the advice of the International Committee of Medical Journal Editors (1991): 'Describe statistical methods with enough detail to enable a knowledgeable reader with access to the original data to verify the reported results'. Poor reporting is one of the failings that is most amenable to improvement. We hope that referees and editors will be more vigilant on this aspect in future.

We also identified many presentational problems. In particular, there was a general tendency to present results as

P-values without quantitative results, and an absence of confidence intervals. Many of the survival plots were of poor quality. Most of the problems were not too serious, but there was considerable scope for improved presentation of information in graphs (see Greenberg *et al.*, 1983). However, the use of sloping lines to join Kaplan–Meier survival curves gives incorrect estimates (Peto *et al.*, 1977). Some of the deficiencies may have been due to limitations of the software used.

We looked at the advice currently given by each of the journals in our survey in their instructions to authors regarding statistical aspects of submitted papers. The *British Journal of Cancer* and the *European Journal of Cancer* make no mention of statistics. *Cancer* and the *Journal of Clinical Oncology* recommend authors to consult the guidelines of Simon and Wittes (1985). The latter also recommends the guidelines of Zelen (1983). The *American Journal of Clinical Oncology* refers to the 'NCI Methodologic Guidelines for Reports of Clinical Trials' published in their journal. Eight of the ten points in this unattributed document (Anonymous, 1986) are in fact taken from Simon and Wittes (1985). The original recommendation about quality control of data was omitted.

The most quoted guidelines (Simon and Wittes, 1985) are cited in some form by three of the five journals in our study (and also by the *Journal of the National Cancer Institute*). Their nine points cover quality control of data; accounting for patients and describing follow-up; rates of ineligibility or missing data; the need for an intention to treat analysis in randomised trials; sample size, power and confidence intervals; planned sample size and reasons for stopping patient accrual; dangers of using non-randomised controls in treatment comparisons and the unacceptability of comparing survival of responders and non-responders; description of patients, extrapolation and subset analyses; and finally a statement of the principle of adequate description already referred to above. Zelen (1983) made a similar set of recommendations, and also discussed other issues including the early reporting of clinical trials. These two sets of sensible guidelines cover many of the issues that we investigated in our review. It is clear that many authors have not taken notice of these recommendations, and that referees and editors have not insisted that authors do so.

These sets of guidelines do not, however, include much advice on the presentation of the results of studies of survival. There seems to be little published advice elsewhere on summarising the data and presenting the results of the analyses, although some authors have considered some of the relevant issues, mostly in the context of the reporting of clinical trials (Greenberg *et al.*, 1983; Meinert, 1986; Gelber and Goldhirsch, 1993). Our findings suggest that guidelines for presentation of survival analysis in medical journals would be useful. Some suggested guidelines are shown in the appendix. These largely follow from the most obvious reporting deficiencies described above. The methodological standard of papers published in cancer journals could be improved if authors were made to adhere to these or similar guidelines for presentation as well as the other guidelines discussed above (Zelen, 1983; Simon and Wittes, 1985).

**Acknowledgements**

We thank David Collett, Robert Edwards, David Machin and Peter Sasieni for comments on an earlier version of the paper.

### References

ALTMAN DG. (1982). Statistics in medical journals. *Stat. Med.*, **1**, 59–71.

ALTMAN DG. (1991). Statistics in medical journals: developments in the 1980s. *Stat. Med.*, **10**, 1897–1913.

ALTMAN DG. (1992). Categorising continuous variables. *Br. J. Cancer*, **64**, 975.

ALTMAN DG, LAUSEN B, SAUERBREI W AND SCHUMACHER M. (1994). Dangers of using 'optimal' cutpoints in the evaluation of prognostic factors. *J. Natl Cancer Inst.*, **86**, 829–835.

ANDERSEN PK. (1991). Survival analysis 1982–91: the second decade of the proportional hazards regression model. *Stat. Med.*, **10**, 1931–1941.

ANDERSON JR, CAIN KC, GELBER RD AND GELMAN RS. (1985). Analysis and interpretation of the comparison of survival by treatment outcome variables in cancer clinical trials. *Cancer Treat. Rep.*, **69**, 1139–1144.

ANONYMOUS. (1986). Methodologic guidelines for reports of clinical trials. *Am. J. Clin. Oncol.*, **9**, 276.

CHRISTENSEN E. (1987). Multivariate survival analysis using Cox's regression model. *Hepatology*, **7**, 1346–1358.

COLLETT D. (1994). *Modelling Survival Data in Medical Research.* Chapman & Hall: London.

COX DR. (1972). Regression models and life-tables (with discussion) *J.R. Stat. Soc. B*, **39**, 86–94.

DAWSON-SAUNDERS B AND TRAPP RG. (1994). *Basic and Clinical Biostatistics*, 2nd edn. Appleton & Lange: East Norwalk, CT.

ELASHOFF JD. (1983). Surviving proportional hazards. *Hepatology*, **3**, 1031–1035.

GELBER RD AND GOLDHIRSCH A. (1992). Reporting and interpreting adjuvant therapy clinical trials. *J. Natl Cancer Inst. Monogr.*, **11**, 59–69.

GØTZSCHE PC. (1989). Methodology and overt and hidden bias in reports of 196 double-blind trials of nonsteroidal, antiinflammatory drugs in rheumatoid arthritis. *Controlled Clin. Trials*, **10**, 31–56.

GREENBERG ER, BARON JA AND COLTON T. (1983). Reporting the results of a clinical trial. In *Clinical Trials: Issues and Approaches*, Shapiro SH and Louis TA. (eds) pp. 191–204. Marcel Dekker: New York.

HARRINGTON DP AND FLEMING TR. (1982). A class of rank test procedures for censored survival data. *Biometrika*, **69**, 553–566.

HAYNES RB, MULROW CD, HUTH EJ, ALTMAN DG AND GARDNER MJ. (1990). More informative abstracts revisited. *Ann. Intern. Med.*, **113**, 69–76.

HOKANSON JA, LUTTMAN DJ AND WEISS GB. (1986). Frequency and diversity of use of statistical techniques in oncology journals. *Cancer Treat. Rep.*, **70**, 589–594.

INTERNATIONAL COMMITTEE OF MEDICAL JOURNAL EDITORS. (1991). Uniform requirements for manuscripts submitted to biomedical journals. *Br. Med. J.*, **302**, 338–341.

MACHIN D AND GARDNER MJ. (1989). Calculating confidence intervals for survival time analyses. In *Statistics with Confidence* Gardner MJ and Altman DG. (eds) pp. 64–70. British Medical Journal: London.

MEINERT C. (1986). Preparation of the study publication. In: *Clinical Trials. Design, Conduct, and Analysis*, pp. 264–270. Oxford University Press: New York.

NELSON W. (1969). Hazard plotting for incomplete failure data. *J. Qual. Technol.*, **1**, 27–52.

PETO R, PIKE MC, ARMITAGE P, BRESLOW NE. *et al.* (1977). Design and analysis of randomized clinical trials requiring prolonged observation of each patient. II. Analysis and examples. *Br. J. Cancer*, **35**, 1–39.

POCOCK SJ, HUGHES MD AND LEE RJ. (1987). Statistical problems in the reporting of clinical trials. A survey of three medical journals. *N. Engl. J. Med.*, **317**, 426–432.

SHUSTER JJ. (1991). Median follow up in clinical trials. *J. Clin. Oncol.*, **9**, 191–192.

SIMON R. (1982). Patient subsets and variation in therapeutic efficacy. *Br. J. Clin. Pharmacol.*, **14**, 473–482.

SIMON R. (1986). Confidence limits for reporting results of clinical trials. *Ann. Intern. Med.*, **105**, 429–435.

SIMON R AND ALTMAN DG. (1994). Statistical aspects of prognostic factor studies in oncology. *Br. J. Cancer*, **69**, 979–985.

SIMON R AND WITTES RE. (1985). Methodologic guidelines for reports of clinical trials. *Cancer Treat. Rep.*, **69**, 1–3.

TIBSHIRANI R. (1982). A plain man's guide to the proportional hazards model. *Clin. Invest. Med.*, **5**, 63–68.

ZELEN M. (1983). Guidelines for published papers on cancer clinical trials: responsibilities of editors and authors. *J. Clin. Oncol.*, **1**, 164–169.

### Appendix:

### Suggested guidelines for presentation of survival analyses

*Presentation of data*

- Describe the recruitment and analysis dates.
- Describe the reason for the sample size.
- Report a summary of follow-up, such as the median and quartiles computed by the reverse Kaplan–Meier method.
- Report how many subjects were lost to follow-up and whether, and how, they had been included in the analyses.
- Report the number of events for each end point.

*Presentation of methods*

- Give a clear definition of each end point being considered, i.e. define the time origin, the event of interest and the circumstances where survival times are censored.
- Name the method used for estimating survival probabilities.
- Name any test used in the analyses; in particular, justify the use of weighted logrank tests.
- Report the test for trend when ordered categorical variables are examined.
- When performing univariate or multivariate analyses, report all the covariates examined, their frequency of missing values and the definition of the categories used (if any) *whether the covariate is significant or not.*
- When Cox regression analyses are performed, describe the criteria used to select the variables in the initial model, the procedure to specify the final model and describe any methods used to assess the model assumptions.
- Name the software used.

*Presentation of results*

- Give a summary of overall survival: preferably median and/or percent surviving *n* years.
- If study is a randomised clinical trial, give separate summaries of survival for each treatment group.
- When reporting the results of any test, give the test statistic, the degrees of freedom (when applicable) and the exact *P*-value.
- When presenting results of a logrank test also report the numbers of observed and expected events in each group (desirable).
- When comparing survival in two or more groups, give an estimate of the survival in each group, e.g. median survival time, survival probabilities for a particular time point, hazard ratio.
- When presenting the results of a Cox regression analysis, report the estimated coefficients (or estimated hazard ratios), measures of their precision (i.e. standard errors or confidence intervals) and/or the associated *P*-values.
- Do not use crude rates to summarise the data.

*Graphs*

- Use meaningful time intervals.
- Use a step function to join Kaplan–Meier survival estimates.
- Mark the survival time of censored observations (desirable).
- If several curves are reported in the same plot use different lines type (desirable).
- Give number of patients at risk at selected time points (desirable).
- Mark confidence intervals or standard errors for some of the selected time points (desirable).

*Abstract*

- Include in the abstract summaries of follow-up and survival (separately by treatment group if applicable) and the final results of both univariate and multivariate analyses (when applicable).